# Supplementary material for: Heteromeric amyloid filaments of ANXA11 and TDP-43 in FTLD-TDP type C
Source: Nature. 2024 Sep 11;634(8034):662–8. doi: 10.1038/s41586-024-08024-5 (PMC11485244; doi:10.1038/s41586-024-08024-5)

nature portfolio

https://doi.org/10.1038/s41586-024-08024-5

Supplementary information

Heteromeric amyloid filaments of ANXA11 and TDP-43 in FTLD-TDP type C

In the format provided by the authors and unedited

Supplementary Figure 1: Immunoblot source data

Fig 4a

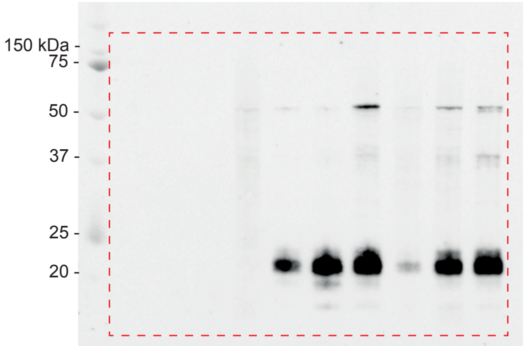

Fig 4b

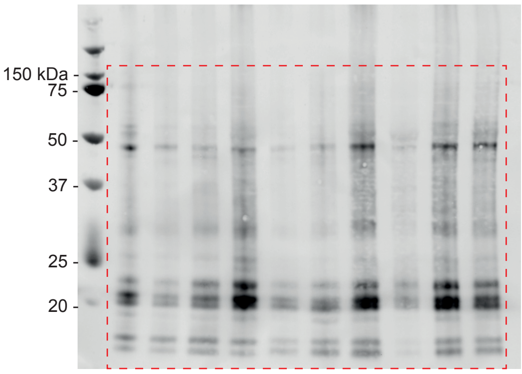

ED Fig 8a

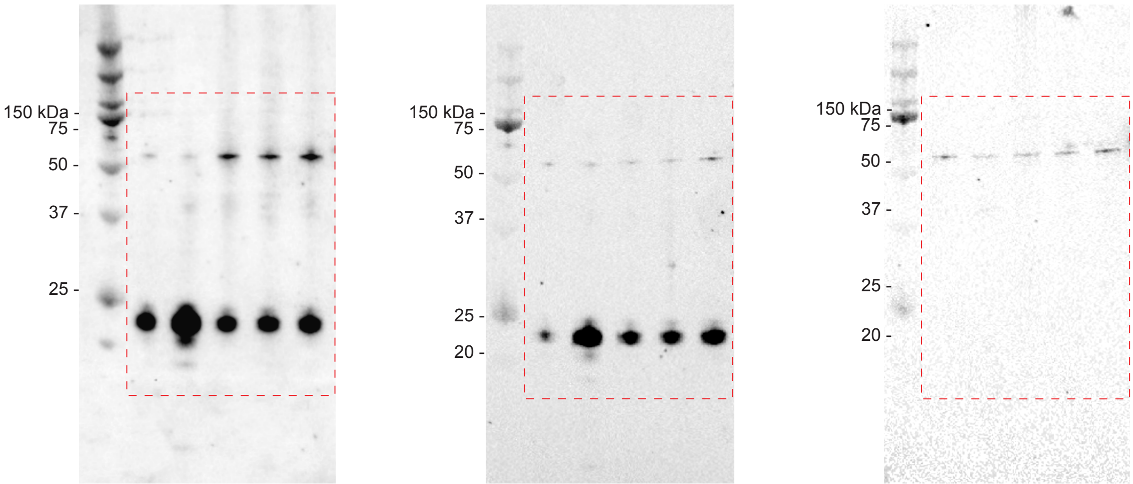

Supplement: Supplementary file 1 — Immunoblot source data. [file 41586_2024_8024_MOESM1_ESM.pdf]
