# Supplementary material for: Heteromeric amyloid filaments of ANXA11 and TDP-43 in FTLD-TDP type C
Source: Nature. 2024 Sep 11;634(8034):662–8. doi: 10.1038/s41586-024-08024-5 (PMC11485244; doi:10.1038/s41586-024-08024-5)
Supplement: Supplementary file 2 — Reporting Summary [file 41586_2024_8024_MOESM2_ESM.pdf]

nature portfolio

Corresponding author(s): Benjamin Ryskeldi-Falcon

Last updated by author(s): Aug 12, 2024

Reporting Summary

Nature Portfolio wishes to improve the reproducibility of the work that we publish. This form provides structure for consistency and transparency in reporting. For further information on Nature Portfolio policies, see our Editorial Policies and the Editorial Policy Checklist.

Statistics

For all statistical analyses, confirm that the following items are present in the figure legend, table legend, main text, or Methods section.

n/a	Confirmed	
☐	☒	The exact sample size (*n*) for each experimental group/condition, given as a discrete number and unit of measurement
☐	☒	A statement on whether measurements were taken from distinct samples or whether the same sample was measured repeatedly
☒	☐	The statistical test(s) used AND whether they are one- or two-sided<br>*Only common tests should be described solely by name; describe more complex techniques in the Methods section.*
☒	☐	A description of all covariates tested
☒	☐	A description of any assumptions or corrections, such as tests of normality and adjustment for multiple comparisons
☐	☒	A full description of the statistical parameters including central tendency (e.g. means) or other basic estimates (e.g. regression coefficient) AND variation (e.g. standard deviation) or associated estimates of uncertainty (e.g. confidence intervals)
☒	☐	For null hypothesis testing, the test statistic (e.g. *F*, *t*, *r*) with confidence intervals, effect sizes, degrees of freedom and *P* value noted<br>*Give P values as exact values whenever suitable.*
☐	☒	For Bayesian analysis, information on the choice of priors and Markov chain Monte Carlo settings
☒	☐	For hierarchical and complex designs, identification of the appropriate level for tests and full reporting of outcomes
☒	☐	Estimates of effect sizes (e.g. Cohen's *d*, Pearson's *r*), indicating how they were calculated

Our web collection on statistics for biologists contains articles on many of the points above.

Software and code

Policy information about availability of computer code

Data collection	EPU 2.14
Data analysis	Mascot 2.4, Scaffold 4.0, RELION 4.0 and 5.0, CTFFIND 4.1, ModelAngelo 1.0, COOT 0.9.8.2, ISOLDE 1.5, REFMAC 5.8.0387, Servalcat 0.3.0, ChimeraX 1.5, MolProbity 4.5.2.

For manuscripts utilizing custom algorithms or software that are central to the research but not yet described in published literature, software must be made available to editors and reviewers. We strongly encourage code deposition in a community repository (e.g. GitHub). See the Nature Portfolio guidelines for submitting code & software for further information.

Data

Policy information about availability of data

All manuscripts must include a data availability statement. This statement should provide the following information, where applicable:

- Accession codes, unique identifiers, or web links for publicly available datasets
- A description of any restrictions on data availability
- For clinical datasets or third party data, please ensure that the statement adheres to our policy

Whole-exome data have been deposited to the National Institute on Ageing Alzheimer's Disease Data Storage Site (NIAGADS) under accession code NG00107. Mass spectrometry data has been deposited to the Proteomics Identifications (PRIDE) database under accession code PXD055345. Cryo-EM datasets have been deposited to the Electron Microscopy Public Image Archive (EMPIAR) under accession codes EMPIAR-12248 (Individual 1), EMPIAR-12247 (Individual 2), EMPIAR-12246

(Individual 3) and EMPIAR-12245 (Individual 4). Cryo-EM reconstructions have been deposited to the Electron Microscopy Data Bank (EMDB) under accession codes EMD-50628 and EMD-50621 (Individual 1, alternative conformations 1 and 2, respectively), EMD-51358 (Individual 2), EMD-51359 (Individual 3), and EMD-51360 (Individual 4). Atomic models have been deposited to the Protein Data Bank (PDB) under accession codes 9FOR and 9FOF (alternative conformations 1 and 2, respectively).

Research involving human participants, their data, or biological material

Policy information about studies with human participants or human data. See also policy information about sex, gender (identity/presentation), and sexual orientation and race, ethnicity and racism.

Reporting on sex and gender	2 males and 2 females
Reporting on race, ethnicity, or other socially relevant groupings	N/A
Population characteristics	See Extended Data Table 1. Between 59 and 86 years-of-age. No neurodegenerative disease-associated genetic variants. Clinical diagnosis of svPPA. Neuropathological diagnosis of FTLD-TDP Type C.
Recruitment	Selected based on availability and neuropathological examination.
Ethics oversight	Human tissue samples were from the Department of Brain and Neurosciences, Tokyo Metropolitan Institute of Medical Science; the Department of Psychiatry, National Hospital Organization Shimofusa Psychiatric Center; the Department of Neuropathology, Tokyo Metropolitan Institute for Geriatrics and Gerontology; and the Brain Library of the Dementia Laboratory at Indiana University School of Medicine. Their use in this study was approved by the ethical review processes at each institution. Informed consent was obtained from the patients' next of kin.

Note that full information on the approval of the study protocol must also be provided in the manuscript.

Field-specific reporting

Please select the one below that is the best fit for your research. If you are not sure, read the appropriate sections before making your selection.

☒ Life sciences ☐ Behavioural & social sciences ☐ Ecological, evolutionary & environmental sciences

For a reference copy of the document with all sections, see nature.com/documents/nr-reporting-summary-flat.pdf

Life sciences study design

All studies must disclose on these points even when the disclosure is negative.

Sample size	Frontotemporal cortex from 4 individuals with FTLD-TDP Type C. Samples were chosen based on availability and neuropathological examination.
Data exclusions	Pre-established common image classification procedures (Scheres 2012. J. Struc. Biol. 180, 519-530) were employed to select particle images with the highest resolution content in the cryo-EM reconstruction process. Details of the number of selected images are given in Extended Data Table 2.
Replication	All attempts at replication were successful. Three independent biological repeats per experiment where representative data are shown, as described in the main text.
Randomization	Randomisation was not performed. As the samples were limited by brain availability, randomisation would not have reduced any bias in this study.
Blinding	The investigators were not blinded to allocation during experiments and outcome assessment. The perceived risk of detection/performance bias was deemed negligible.

Reporting for specific materials, systems and methods

We require information from authors about some types of materials, experimental systems and methods used in many studies. Here, indicate whether each material, system or method listed is relevant to your study. If you are not sure if a list item applies to your research, read the appropriate section before selecting a response.

Materials & experimental systems

n/a	Involved in the study	
☐	☒	Antibodies
☒	☐	Eukaryotic cell lines
☒	☐	Palaeontology and archaeology
☒	☐	Animals and other organisms
☒	☐	Clinical data
☒	☐	Dual use research of concern
☒	☐	Plants

Methods

n/a	Involved in the study	
☒	☐	ChIP-seq
☒	☐	Flow cytometry
☒	☐	MRI-based neuroimaging

Antibodies

Antibodies used

The primary antibodies used were anti-phospho S409 and 410 TDP-43 (CosmoBio CAC-TIP-PTD-M01A), anti-ANXA11 residues 3–15 (OriGene TA302761), anti-ANXA11 residues 1–180 (Proteintech 10479-2-AP) and anti-ANXA11 residues 276–505 (St John's Laboratory STJ29559).

Validation

Validation of anti-phospho S409 and S410 TDP-43 is presented in the manufacturer's datasheet (Cosmo Bio USA) and in (Inukai et al. 2008. FEBS Lett. 582, 2899-2904). Validation of anti-ANXA11 residues 3–15 is presented in the manufacturer's datasheet (OriGene). Validation of anti-Annexin A11 residues 1–180 is presented in the manufacturer's datasheet (Proteintech) and in (Smith et al. 2017. Sci Transl Med 9, eaad9157). Validation of anti-ANXA11 residues 276–505 is presented in the manufacturer's datasheet (St John's Laboratory).

Plants

Seed stocks

Report on the source of all seed stocks or other plant material used. If applicable, state the seed stock centre and catalogue number. If plant specimens were collected from the field, describe the collection location, date and sampling procedures.

Novel plant genotypes

Describe the methods by which all novel plant genotypes were produced. This includes those generated by transgenic approaches, gene editing, chemical/radiation-based mutagenesis and hybridization. For transgenic lines, describe the transformation method, the number of independent lines analyzed and the generation upon which experiments were performed. For gene-edited lines, describe the editor used, the endogenous sequence targeted for editing, the targeting guide RNA sequence (if applicable) and how the editor was applied.

Authentication

Describe any authentication procedures for each seed stock used or novel genotype generated. Describe any experiments used to assess the effect of a mutation and, where applicable, how potential secondary effects (e.g. second site T-DNA insertions, mosiacism, off-target gene editing) were examined.
